# Overexpression of G-Protein-Coupled Receptor 40 Enhances the Mitogenic Response to Epoxyeicosatrienoic Acids

**DOI:** 10.1371/journal.pone.0113130

**Published:** 2015-02-13

**Authors:** Seong Kwon Ma, Yinqiu Wang, Jianchun Chen, Ming-Zhi Zhang, Raymond C. Harris, Jian-Kang Chen

**Affiliations:** 1 Department of Medicine, Vanderbilt University School of Medicine, Nashville, Tennessee, United States of America; 2 Department of Internal Medicine, Chonnam National University Medical School, Gwangju, Korea; 3 Departments of Cellular Biology & Anatomy and Medicine, Medical College of Georgia, Georgia Regents University, Augusta, Georgia, United States of America; Emory University, UNITED STATES

## Abstract

The cytochrome P450 epoxygenase-dependent arachidonic acid metabolites, epoxyeicosatrienoic acids (EETs), are potent survival factors and mitogens for renal epithelial cells, but the molecular identity in the cells that initiates the mitogenic signaling of EETs has remained elusive. We screened kidney cell lines for the expression of G-protein-coupled receptor 40 (GPR40) and found that the porcine renal tubular epithelial cell line LLCPKcl4, which has been previously demonstrated to be sensitive to the mitogenic effect of EETs, expresses higher levels of GPR40 mRNA and protein than the human embryonic kidney cell line HEK293. EETs induced only a weak mitogenic EGFR signaling and mild cell proliferation in HEK293 cells. To determine whether GPR40 expression level is what mediates the mitogenic sensitivity of cells to EETs, we created a human GPR40 (hGPR40) cDNA construct and transfected it into HEK293 cells and picked up a number of stable transfectants. We found that GPR40 overexpression in HEK293 cells indeed significantly enhanced EET-induced cell proliferation and markedly augmented EGFR phosphorylation ERK activation, which were inhibited by the EGFR tyrosine kinase inhibitor, AG1478, or the HB-EGF inhibitor, CRM197. EETs significantly enhanced release of soluble HB-EGF, a natural ligand of EGFR, into the culture medium of hGPR40-transfected HEK293 cells, compared to empty vector-transfected cells. In mouse kidneys, markedly higher level of GPR40 protein was found in the cortex and outer stripe of outer medulla compared to the inner stripe of outer medulla and inner medulla. In situ hybridization confirmed that GPR40 mRNA was localized to a subset of renal tubules in the kidney, including the cortical collecting duct. Thus, this study provides the first demonstration that upregulation of GPR40 expression enhances the mitogenic response to EETs and a relatively high expression level of GPR40 is detected in a subset of tubules including cortical collecting ducts in the mammalian kidney.

## Introduction

Arachidonic acid is a polyunsaturated 20 carbon omega-6 fatty acid that is an important constituent of cellular membranes. It is normally esterified to the *sn*-2 position of glycerophospholipids and is released from selected lipid stores following activation of specific phospholipases [1]. Metabolism of released arachidonic acid by the cytochrome P450-dependent monooxygenase pathway produces biologically active compounds in two ways: epoxidation, producing 5,6-, 8,9-, 11,12-, 14,15-epoxyeicosatrienoic acids (EETs) and ω-/ω-1 hydroxylation, resulting in the formation of 19- and 20-hydroxyeicosatetraenoic acids (HETEs) [2–4]. EETs have been demonstrated to play important roles in regulating vascular tone, platelet aggregation, tissue and body homeostasis, Ca^2+^ signaling and steroidogenesis [3–9]. In addition, EETs have been suggested to be an endothelium-derived hyperpolarizing factor in vasculature [10,11].

EETs are synthesized primarily by epoxygenases of the 2C gene subfamily of cytochrome P450 enzymes (Cyp2c) that are highly expressed in the mammalian kidney. Our previous studies indicate that EETs promote renal epithelial cell survival by activation of a PI3K-Akt signaling pathway [15]. We also reported that EETs stimulate proliferation of cultured renal epithelial cells and elucidated underlying intracellular signaling pathways [12–14, 16]. More recent studies have demonstrated that Cyp2c44 epoxygenase is responsible for the production of EETs that activate ERK1/2 to phosphorylate and inhibit the γ subunit of the epithelial sodium channel (ENaC) in the kidney; therefore, knocking out Cyp2c44 epoxygenase causes dietary salt-sensitive hypertension [17]. However, these prior studies did not pinpoint the site that EETs act upon to initiate the intracellular signaling cascades and did not identify a functional receptor for EETs.

G-protein-coupled receptor 40 (GPR40) was first described as an orphan 7-transmembrane G protein-coupled receptor in 1997 [18]. Subsequent studies identified medium- and long-chain fatty acids as its putative ligands even at physiologically relevant concentrations. Accordingly, the receptor is now also known as free fatty acid receptor 1 (FFAR1), the first identified member of a family of FFARs that also includes GPR41, GPR43, GPR119 and GPR120 [19]. Of note, GPR40 is most highly expressed in brain and pancreatic islets, leading to the suggestion that GPR40 is involved in the regulation of insulin secretion in response to free fatty acids [20,21]. Interestingly, subsequent studies increasingly indicate that GPR40 is also expressed in other organs [20–22]. To date, however, no previous studies have investigated the expression and function of GPR40 in the mammalian kidney.

Here we show that during a screening for GPR40 expression in kidney cell lines, we found that the porcine renal tubular epithelial cell line LLCPKcl4, which is sensitive to EETs and has been utilized to identify the biological effects and the underlying molecular signaling mechanism of EETs [12–16], expresses a markedly higher level of GPR40 than the human embryonic kidney cell line HEK293, which we confirmed is not sensitive to the mitogenic response to EETs. We therefore created a human GPR40 (hGPR40) cDNA construct and transfected it into the HEK293 cells and established several stable clones expressing hGPR40 to test whether the sensitivity of cells to the mitogenic signaling and biological response to EETs is mediated by GPR40. We also examined the expression pattern of GPR40 in mammalian kidney. Our results suggest that upregulation of GPR40 expression enhances the release of soluble HB-EGF, which serves as a natural ligand to transactivate EGF receptor and downstream ERK phosphorylation and biological effects of EETs. We also determined that even under the normal physiological condition, GPR40 is indeed expressed in the nephron segments of the mammalian kidneys, including branching cortical collecting ducts.

## Results

**Fig 1:**
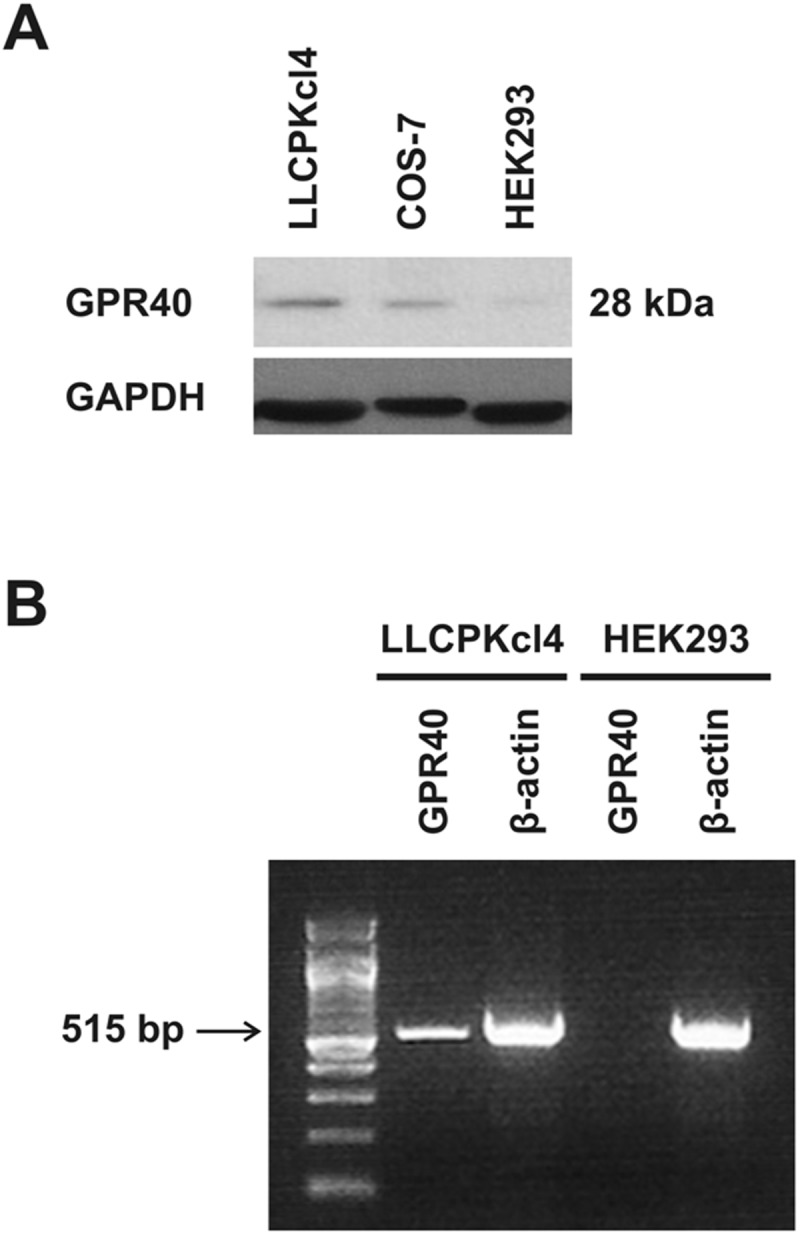
GPR40 expression in different cell lines. (A) The protein expression of GPR40 was most abundant in the renal tubule epithelial cell line LLCPKcl4 but very low in HEK293 cells, as revealed by immunoblotting a rabbit anti-human GPR40 antibody (Epitomics, Burlingame, CA) that also cross-reacts with monkey, porcine, and mouse GPR40. Shown was a representative blot from at least three separate experiments with similar results. (B) RT-PCR demonstrated that GPR40 mRNA expression was higher in LLCPKcl4 cells, with minimal expression in HEK293 cells.

**Fig 2:**
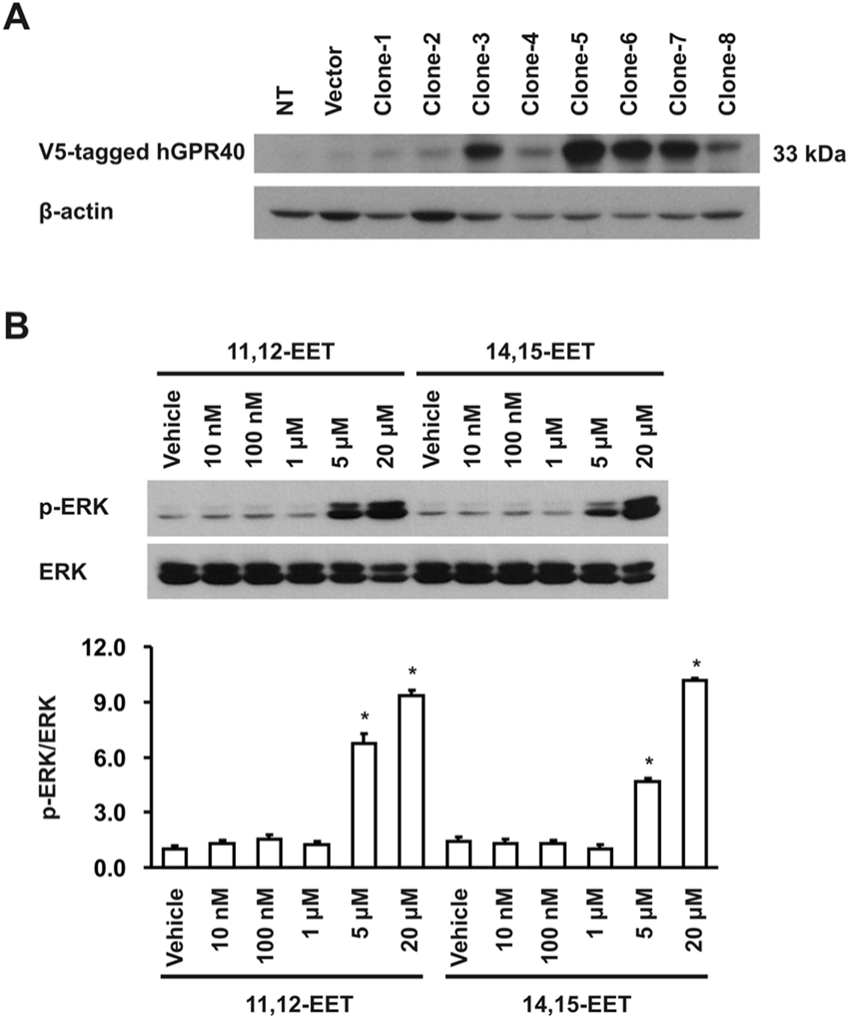
(A) The protein expression of human GPR40 (hGPR40) in the HEK293 cells transfected with human GPR40 cDNA construct. An anti-human GPR40 antibody was used for immunoblotting. Shown are representative stable clones expressing different levels of human GPR40. NT, non-transfected HEK293 cells, used as a negative control. (B) Quiescent hGPR40-transfected HEK293 cells were exposed to different concentrations of 11,12-EET or 14,15-EET for 15 min. 11,12-EET and 14,15-EET concentration-dependently induced ERK1/2 phosphorylation, respectively, in hGPR40-transfected HEK293 cells, compared with vehicle. The same immunoblot was stripped and re-probed with total ERK antibodies to ensure equal loading and transfer. Shown are representative blots of 3 separate experiments with similar results. *p<0.05 compared with vehicle alone in hGPR40 transfected HEK293 cells.

**Fig 3:**
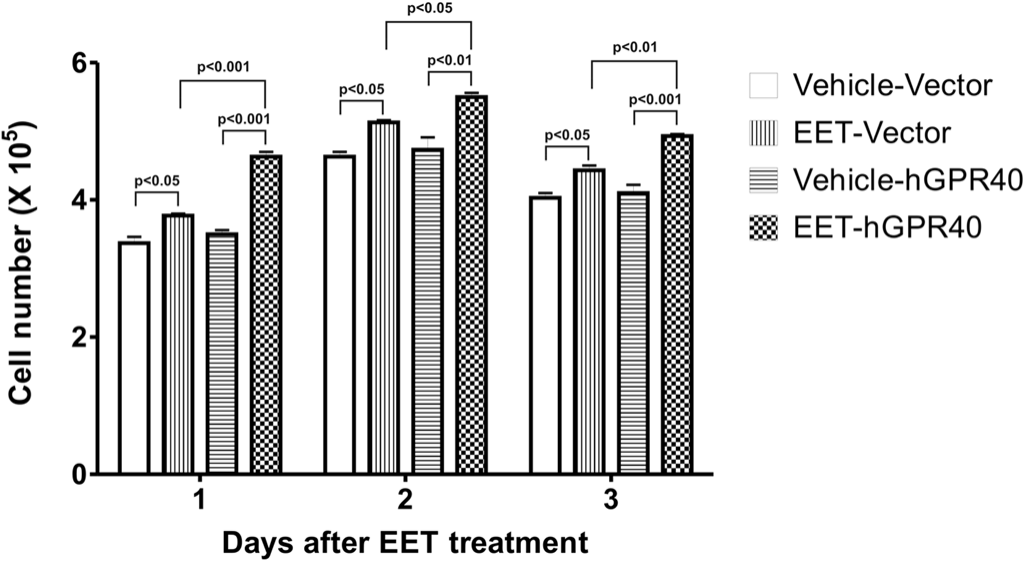
Effect of human GPR40 transfection on EET-induced cell proliferation. 1.5 × 10^5^/well HEK293 cells transfected with hGPR40 or empty-vector were seeded into 24-well plates, respectively. 24 h later, the cells were made quiescent in serum-free medium, followed by stimulated with 5 μM 14,15-EET or vehicle alone in the presence of 0.5% FBS in the medium. Cell number per well in each treatment group was counted every day. Results were plotted as the cell number/well for the indicated durations. Each experimental data point represents triplicate wells from three different experiments. Human GPR40 overexpression in HEK293 cells significantly augmented 14,15-EET-stimulated cell proliferation, compared with empty vector-transfected HEK293 cells.

In initial studies, we screened kidney epithelial cell lines for GPR40 expression, and found that the porcine renal tubular epithelial cell line LLCPKcl4 expressed markedly higher levels of GPR40 mRNA and protein while the human embryonic kidney cell line HEK293 had a very low level of GPR40 expression (Fig. 1) and confirmed that HEK293 cells are not sensitive to the mitogenic effect of EETs. Previous studies revealed that LLCPKcl4 cells are sensitive to the mitogenic effect of EETs and have been utilized to identify the tyrosine kinase phosphorylation cascade activated by EETs [12–16]. To test whether increasing the expression level of GPR40 in the HEK293 cells could increase their sensitivity to the mitogenic effect of EETs, we designed a pair primer specific for hGPR40 cDNA and generated a human GPR40 (hGPR40) cDNA construct utilizing the mammalian expression vector, pcDNA3.1/V5-His-TOPO vector as detailed in *Methods*. We then transfected the hGPR40 cDNA construct into HEK293 cells and developed a number of HEK293 cell clones stably expressing human GPR40 (Fig. 2A). The stable HEK293 clones overexpressing hGPR40 showed markedly increased ERK phosphorylation in response to 11,12-EET and 14,15-EET in a concentration-dependent manner. Shown in Fig. 2B are representative results from clone-5. These experiments were repeated in at least two of these stable clones with similar results. Importantly, 14,15-EET-stimulated cell proliferation was significantly augmented in these hGPR40-overexpressing stable clones, compared to the empty vector-transfected HEK293 cells, which showed a small but significant increase in cell number in response to 14, 15-EET stimulation (Fig. 3), consistent with the low basal expression level of GPR40 protein in non-transfected HEK293 cells (Fig. 1A).

**Fig 4:**
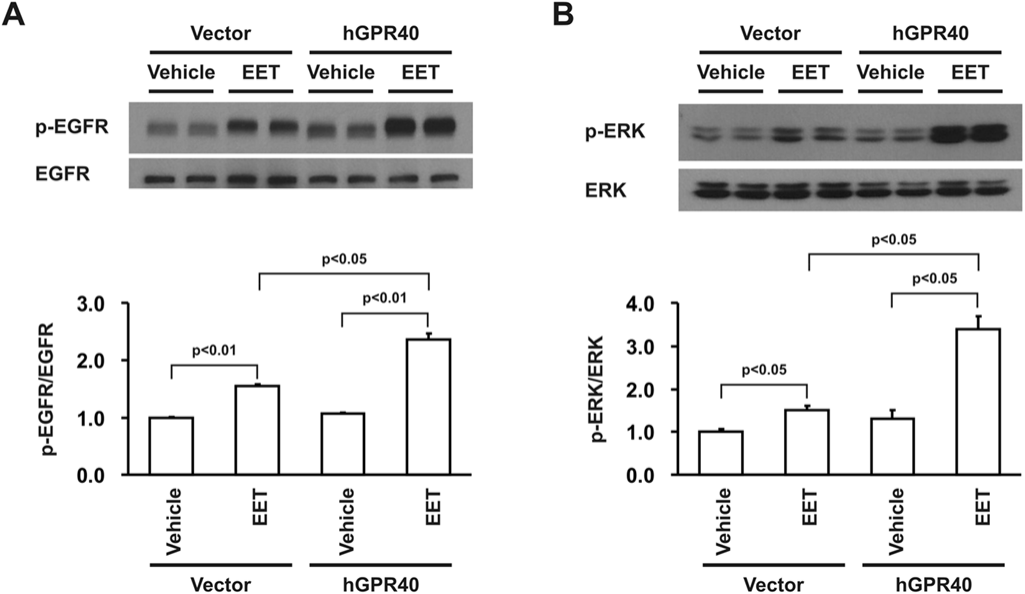
Effect of human GPR40 transfection on EET-induced phosphorylation of EGFR and ERK in HEK293 cells. Compared to empty vector-transfected HEK293 cells, the clones stably overexpressing human GPR40 exhibited markedly augmented phosphorylation of both EGFR and ERK in response to 14,15-EET (5 μM for 15 min). Shown are representative blots of 3 separate experiments with similar results and the quantitative analysis by densitometry from 3 independent experiments.

**Fig 5:**
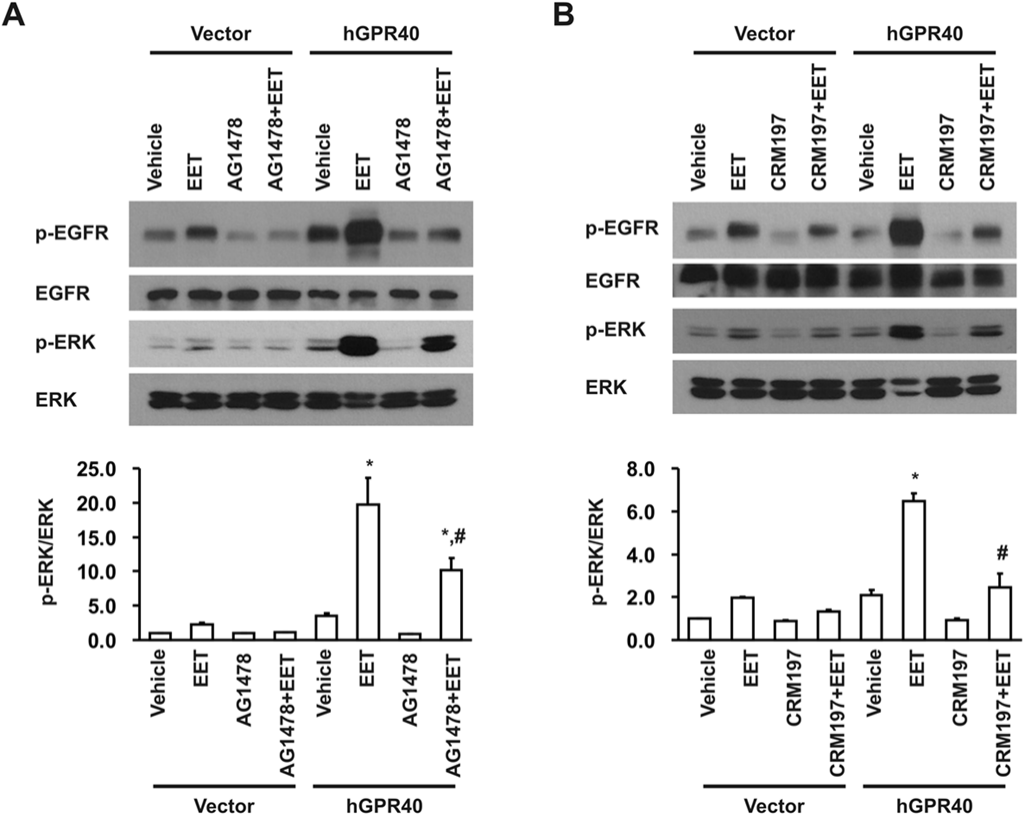
Effect of EGFR or HB-EGF inhibition on EET-stimulated phosphorylation of EGFR and ERK in HEK293 cells. Quiescent HEK293 cells were treated with 5 μM 14,15-EET for 15 min. Human GPR40 (hGPR40) overexpression markedly augmented EET-induced phosphorylation of EGFR and ERK, which were inhibited by pretreatment of the cells with the EGFR tyrosine kinase inhibitor, AG1478 (A) or the HB-EGF inhibitor, CRM197 (B). Shown are representative blots from 1 of 3 separate experiments with similar results. *p<0.05 compared with vehicle alone in hGPR40 transfected HEK293 cells. #*p*<0.05 compared with 14,15-EET treated cells in hGPR40 transfected HEK293 cells.

**Fig 6:**
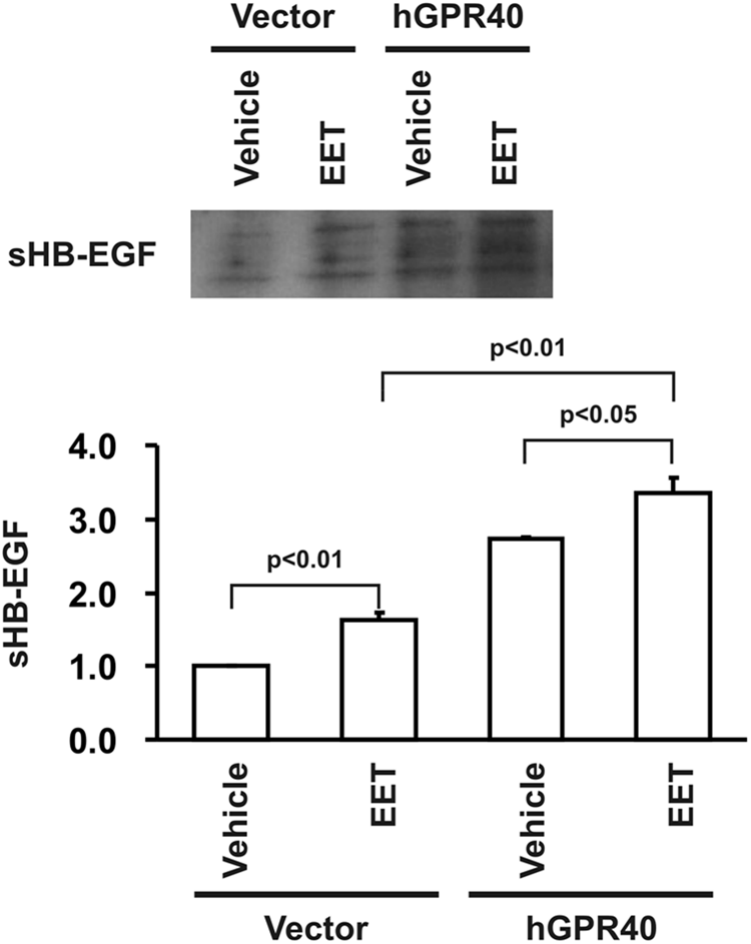
Overexpression of human GPR40 in HEK293 cells enhanced release of soluble HB-EGF into conditioned media. Empty vector-transfected HEK293 cells (Vector) and human GPR40-transfected HEK293 cells (hGPR40) were rendered quiescent in serum-free medium and washed twice with PBS, followed by treatment with vehicle or 14,15-EET, respectively. The conditioned media were applied to a heparin-Sepharose column to purify heparin-binding proteins as described in Experimental Procedures and analyzed by immunoblotting with an antibody specific for HB-EGF. Shown is a representative blot from three separate experiments with similar results and the densitometry quantification of released soluble HB-EGF from the three experiments.

Previous studies demonstrated that EET-dependent cell proliferation in porcine renal tubular epithelial cells is mediated by EGFR transactivation and ERK activation [16]. In the present study, we observed minimal EGFR and ERK phosphorylation in response to 14,15-EET in empty vector-transfected HEK293 cells (Fig. 4), consistent with the minimal expression of GPR40 protein in the non-transfected HEK293 cells (Fig. 1A). In contrast, 14,15-EET-induced phosphorylation of both EGFR and ERK were markedly augmented in GPR40-transfected HEK293 cells (Fig. 4). To confirm that the observed increases in ERK phosphorylation were mediated by EGFR activation, we pretreated the cells with the EGFR tyrosine kinase inhibitor, AG1478, and found that the EET-induced ERK phosphorylation was markedly inhibited by AG1478 inhibition of EGFR phosphorylation (Fig. 5A). Previous studies demonstrated that EET-stimulated EGFR transactivation was mediated by cleavage of membrane-bound HB-EGF and release of soluble HB-EGF (sHB-EGF), a natural ligand for EGFR [16]. Accordingly, we pretreated the GPR40-overexpressing cells with the HB-EGF inhibitor, CRM197. As indicated in Fig. 5B, CRM197 pretreatment inhibited the EET-induced increases in EGFR and ERK phosphorylation. Furthermore, we also measured sHB-EGF in the culture medium of HEK293 cells in response to 14,15-EET administration and detected significantly more sHB-EGF release into the culture medium of GPR40-overexpressing HEK293 cells in response to EET stimulation (Fig. 6). These data suggest that increasing GPR40 expression indeed enhanced the sensitivity of HEK293 cells to the mitogenic stimulation of EETs by augmenting sHB-EGF release and EGFR transactivation-dependent ERK phosphorylation.

**Fig 7:**
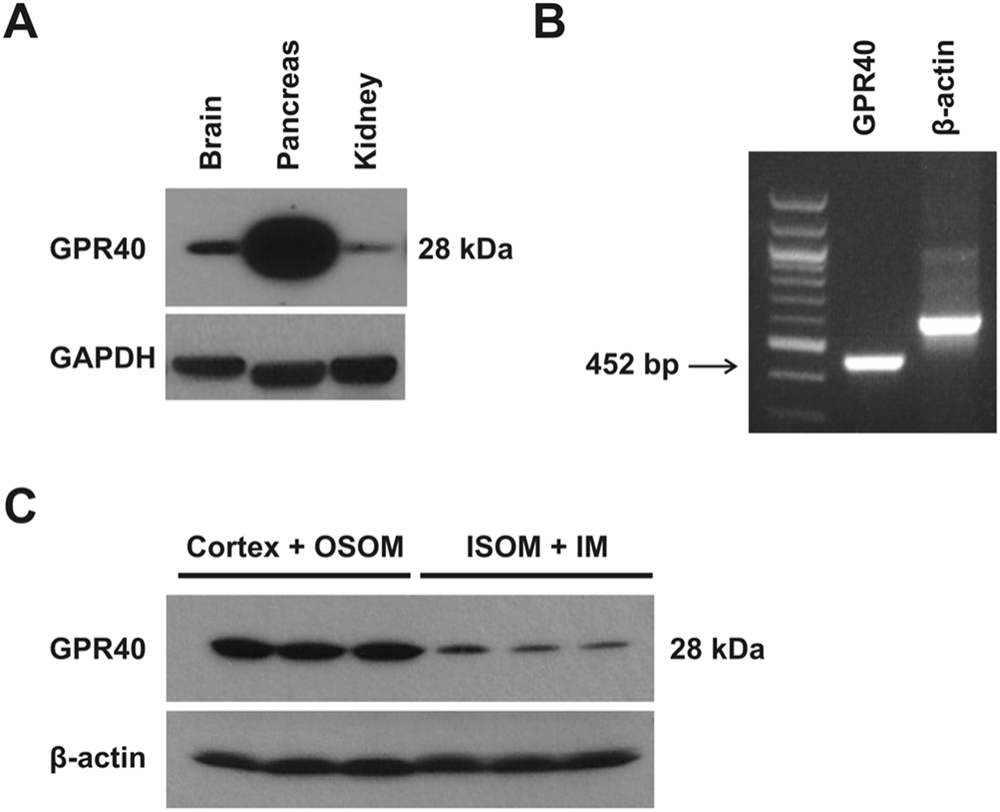
GPR40 expression in murine kidney. (A) In addition to pancreas and brain (used as positive controls), kidneys also express GPR40 in the mouse, revealed by similar immunoblotting analysis. B) RT-PCR indicated GPR40 mRNA was expressed in mouse kidneys. (C) Immunoblotting revealed that the levels of GPR40 protein were higher in the cortex and outer stripe of outer medulla (OSOM) than in inner stripe of outer medulla (ISOM) and inner medulla (IM). Similar results were obtained using two antibodies: one from Epitomics (Burlingame, CA) and another from Santa Cruz Biotechnology (Santa Cruz, CA). Both were rabbit antibodies raised against GPR40 of human origin but according to manufacturer’s data sheets, both antibodies are recommended for the detection of GPR40 of human, mouse, and rat origins. Shown in (A) and (C) are representative immunoblots resulted from the GPR40 antibody from Epitomics.

**Fig 8:**
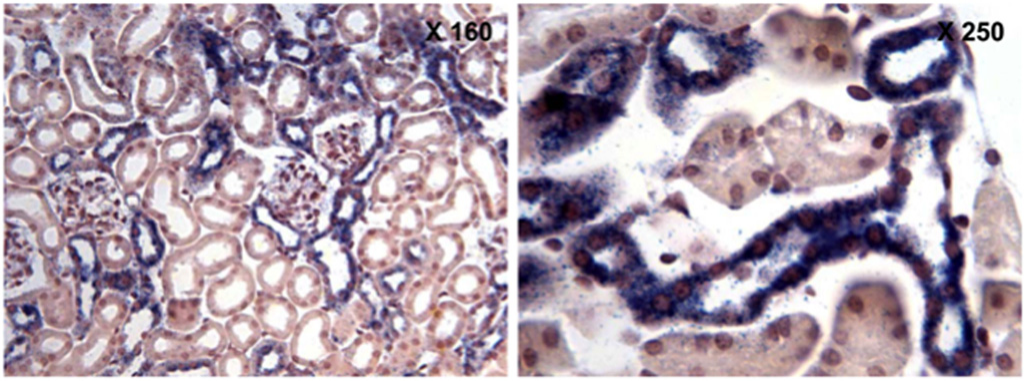
Localization of GPR40 mRNA in mouse kidney. In situ hybridization showed that GPR40 mRNA is expressed in the renal cortical tubules. Note the branching indicating expression in cortical collecting duct.

In addition to cultured renal epithelial cells, we determined that GPR40 mRNA is indeed expressed in mouse kidney (Fig. 7). Immunoblotting indicated that the protein expression levels of GPR40 were higher in cortex and OSOM than ISOM and inner medulla (Fig. 7C). In situ hybridization revealed that GPR40 mRNA was localized to a subset of renal tubules in the kidney cortex, including the collecting duct (Fig. 8).

## Discussion

EETs are produced predominantly by epoxygenases of the 2C gene subfamily of cytochrome P450s in the mammalian kidney [25–29]. Utilizing the EET-sensitive porcine renal epithelial cell line LLCPKcl4, we have previously demonstrated that EETs stimulates cell proliferation by releasing the natural EGFR ligand, sHB-EGF and thus activating EGFR-dependent ERK signaling pathway [12–16]. The present studies indicate that the medium- and long-chain fatty acid receptor GPR40 is expressed in renal epithelia of the mammalian kidney and this EET-sensitive LLCPKcl4 cell line expresses a relatively higher level of GPR40 than the HEK293 cell line. By increasing GPR40 expression in HEK293 cells, we were able to sensitize HEK293 cells to the mitogenic signaling and biological effect of EETs. Specifically, in the presence of upregulated GPR40 expression in cultured renal epithelial cells, EET induces EGFR activation and downstream signaling by releasing soluble HB-EGF, which is a natural ligand for EGF receptor. The released HB-EGF binds to EGFR and activates its intrinsic receptor tyrosine kinase and subsequent ERK activation. These data also provide evidence that inhibiting activity of soluble HB-EGF or blocking the intrinsic EGFR tyrosine kinase is each sufficient to inhibit GPR40-mediated intracellular signaling initiated by EETs.

A previous study indicated that the fatty acid, oleic acid, activated EGFR signaling in breast cancer cells. Oleic acid can serve as a ligand for GPR40, as well as the related fatty acid receptor GPR120, and MCF-7 cells, the cell type used in the studies, expressed both receptors [30]. However, the studies did not directly test whether GPR40 activation was necessary for the EGFR activation in these cells. Since its deorphanization and identification as a receptor for medium- and long-chain fatty acids in 2003, there has been increasing interest in defining the potential physiologic roles of GPR40 [20]. The majority of attention has been directed at possible roles in regulation of insulin secretion. In this regard, GPR40 agonists have been developed as possible agents to stimulate insulin secretion in type 2 diabetes [31–33]. GPR40 is also expressed in enteroendocrine cells of gastrointestinal tract, and may mediate release of glucagon-like peptide-1 and cholecystokinin secretion [34,35]. In addition, GPR40 is expressed in murine skin and may serve to limit and attenuate inflammation [36]. Recent studies have also implicated GPR40 in regulation of pain perception [37]. Finally, GPR40, along with another fatty acid receptor, GPR120, has been implicated in sensing taste of fatty acids [38].

However, renal expression and function of GPR40 have not yet been investigated. In the present study, we demonstrated that overexpression of GPR40 enhances mitogenic signaling of EETs, cytochrome P450 epoxygenase metabolites of the 20-carbon fatty acid, arachidonic acid. We have also determined that a relatively high expression level of GPR40 is detected in a subset of kidney tubules, including the cortical collecting duct, the nephron segment that can branch, in the mammalian kidney. Expression of GPR40 in cortical collecting duct (CCD) is of interest because recent studies indicate that a potential physiologic role for EETs is to modulate sodium reabsorption in this nephron segment by inhibiting epithelial sodium channel (ENaC) open probability [28]. Furthermore, previous studies demonstrated that EGFR activation inhibited CCD sodium reabsorption [39,40]. Our recent study also indicated a role for EGFR activation in EET-mediated ENaC inhibition [41]. Given the identification of GPR40 expression in CCD in the current studies, it is interesting to speculate that it may play a role in modulation of ENaC activity and sodium reabsorption, but further studies will be necessary to determine physiologic roles of GPR40 in mammalian kidney.

In summary, our studies revealed that EETs can induce mitogenic signaling by inducing EGFR transactivation through release of the EGFR ligand, HB-EGF in a GPR40-dependent manner. We further demonstrated that GPR40 is expressed in renal epithelia in vivo. However, additional experiments are required to determine whether GPR40 serves as a receptor to mediate the biologic effects of EETs.

## Materials and Methods

### Reagents and antibodies

Anti-EGFR and anti-phospho-EGFR antibodies were purchased from Cell Signaling Technology (Beverly, MA). Anti-ERK and anti-phospho-ERK antibodies were from Santa Cruz Biotechnology (Santa Cruz, CA). An antibody specific to heparin-binding epidermal growth factor-like growth factor (HB-EGF) was from R&D Systems (Minneapolis, MN). Anti-GPR40 antibodies were obtained from Epitomics (Burlingame, CA) and Santa Cruz Biotechnology (Santa Cruz, CA). Fluorescein Lotus tetragonolobus agglutinin (LTA) was from Vector Laboratories (Burlingame, CA). Anti-aquaporin-2 (AQP2) antibody was from Abcam (Cambridge, MA). Anti-Tamm-Horsfall protein (THP) antibody was from AbD Serotec (Raleigh, NC). Anti-calbindin-D28K antibody was from Sigma-Aldrich (St. Louis, MO). EETs were a generous gift from Jorge Capdevila (Vanderbilt University). Heparin-Sepharose CL-6B column was from Amersham Pharmacia Biotech (Piscataway, NJ). AG1478 was purchased from Calbiochem (San Diego, CA). CRM197 and all other chemicals were from Sigma-Aldrich (St. Louis, MO).

### Cell culture

The human embryonic kidney cell line HEK293 (ATCC), the African green monkey kidney cell line COS-7 (ATCC), and the porcine renal tubular epithelial cell line LLCPKcl4 (obtained from Lee Limbird; originally developed by Kurt Amsler) were routinely cultured in Dulbecco’s modified Eagle’s medium (DMEM)/F-12 mixture supplemented with 10% fetal bovine serum (FBS) at 37°C in a 5% CO_2_ cell culture incubator. The medium was changed every 2 to 3 days.

### Animal Care and Usage

Animals were housed at the Vanderbilt veterinary facility. Animal care and all experimental protocols in our studies were approved by the Vanderbilt University Institutional Animal Use and Care Committee, and all experiments were conducted according to the regulations of Vanderbilt University’s Institutional Animal Care and Usage Committee and complied with the guidelines of National Institutes of Health. Eight weeks old male Balb/c mice from The Jackson Laboratory (Bar Harbor, ME) were euthanized and their kidneys, pancreas, and brains were harvested to determine the expression of GPR40.

### RT-PCR

Total RNA was extracted by using TRI-reagent (Molecular Research Center, Cincinnati, OH) and chloroform extraction [12]. Three micrograms of total RNA was used for reverse-transcription utilizing the First Strand cDNA Synthesis Kit from Amersham Pharmacia Biotech (Piscataway, NJ). The resultant first-strand cDNA was then amplified using primer pairs specific for GPR40 in a GeneAMP 9600 PCR System using Taq polymerase (PerkinElmer/Cetus, Boston, MA). PCR was performed for 30 cycles at 95°C for 45 s, 65°C for 45 s, and 72°C for 60 s followed by a 7 min extension at 72°C. The primer pairs used for the amplification of porcine GPR40 from LLCPKcl4 cells are: 5`-GCT GCT CTG ACC TCC TGC TGG-3’ (forward) and 5’-TAG CTT CCG TCT GTG GCT CAG G-3’ (reverse). For amplification of human GPR40 from HEK293 cells, we used 5’-ATG GAC CTG CCC CCG CAG CTC-3’ (forward primer) and 5’-CTT CTG GGA CTT GCC CCC TTG-3’ (reverse primer).

### Construction of human GPR40 expression vector

To stably transfect human GPR40 cDNA into HEK293 cells, the cDNA containing the entire coding region of human GPR40 was amplified with a synthetic primer pair (Sense: GCC ACC ATG GAC CTG CCC CCG CAG CTC and antisense: CTT CTG GGA CTT GCC CCC TTG CGT TC) utilizing high-fidelity-Taq PCR with a cDNA clone containing the full length of human GPR40 (FFAR1) cDNA as template (Thermo Scientific Open Biosystems, Huntsville, AL). The amplification product was purified and subcloned into the mammalian expression vector, pcDNA3.1/V5-His-TOPO vector (Invitrogen, Carlsbad, CA). The expected sense orientation to the human cytomegalovirus (CMV) promoter in the expression vector was identified by restriction enzyme digestion analysis, and the correct sequence of human GPR40 cDNA construct was further confirmed by sequencing analysis.

### Transfection

Stable transfection of the human GPR40 cDNA construct or empty vector alone (used as a control) into HEK293 cells, respectively, and selection of stable transfectants expressing human GPR40 were conducted as described previously [13].

### Cell proliferation

The assay was performed as we have previously described [12,13]. Briefly, a total of 1.5 × 10^5^ HEK293 cells per well were seeded into 24-well plates. After 24 h of growth in DMEM/F-12 mixture containing 10% FBS, the medium was changed to serum-free medium and incubated for an additional 48 h to make the cells quiescent. Cells were then exposed to 5 μM EET or vehicle alone in the presence of 0.5% FBS in the medium. Cell number per well in each treatment group was counted every day. Results were plotted as the cell number/well for the indicated durations. Each experimental data point represents triplicate wells from three different experiments.

### Immunoblotting

For in vitro experiments, cells were made quiescent in serum free medium for 48 hours before treatment of the cells with the indicated reagents. The cells were then washed twice with ice-cold Ca^2+^/Mg^2+^-free phosphate-buffered saline (PBS) and lysed on ice for 30 min with RIPA buffer [12]. For in vivo experiments, the renal cortex along with the outer strip of outer medulla (OSOM) were dissected and the inner stripe of outer medulla (ISOM) along with the inner medulla of mouse kidneys were homogenized in RIPA buffer. Lysates were clarified by centrifugation at 10,000 × g for 15 min at 4°C, and protein concentrations were determined. The lysates were subjected to 7.5–15% sodium dodecyl sulfate (SDS)-polyacrylamide gels, transferred onto PVDF membranes, probed with the indicated primary antibody and the appropriate secondary antibody conjugated with biotin, incubated with preformed avidin-biotin-horseradish peroxidase complex using a commercially available kit (Vectastatin ABC kit, Vector laboratories, Burlingame, CA), and detected by a peroxidase-catalyzed enhanced chemiluminescence detection system (PerkinElmer, Boston, MA).

### Purification of secreted HB-EGF

Secreted soluble HB-EGF (sHB-EGF) was purified by a modification of previously described heparin affinity chromatography [23]. Briefly, before exposure to vehicle or the indicated agents in serum-free media, confluent cells were rendered quiescent and washed twice with PBS. Conditioned media (CM) were centrifuged and filtered through a 0.45-mm filter. Each sample of CM was immediately applied to a heparin-sepharose column which was preequilibrated with 10 mM Tris-HCl (pH 7.4) containing 0.2 M NaCl and 1 mM benzamidine. After extensive washing with equilibration buffer, bound proteins were eluted with 2.0 M NaCl in 10 mM Tris-HCl (pH 7.4) and dialyzed against 10 mM Tris-HCl (pH 7.4) at 4°C overnight. The dialyzed effluent was lyophilized, then resuspended and boiled in protein sample buffer before separation on 18% SDS-polyacrylamide gels, followed by immunoblotting with an anti-HB-EGF antibody [16].

### In situ hybridization

In situ hybridization was performed with digoxigenin-labeled nucleic acid probes as described previously with some modifications [24]. Briefly, the mouse GPR40 gene antisense probe was labeled with DIG RNA labeling kit (Roche Applied Science, Mannheim, Germany) and the sense probe was synthesized at the same time as a control. Mouse kidneys were fixed in 4% paraformaldehyde, then processed to 10 μm paraffin sections. Paraffin slides were fixed in 4% paraformaldehyde for 10 min, deparaffinized and deproteinized with protease K for 15 min. Slides were then fixed again with 4% paraformaldehyde for 5 min. After washing with PBS, slides were acetylated for 10 min, and permeabilized with 1% Triton X-100 for 20 min. After washing with PBS, pre-hybridization was carried out at 55°C for 2 h. Subsequently, slides were incubated in hybridization buffer with probes at 55°C overnight and then washed with 0.2X SSC for 2 h, then Tris-Saline buffer for 5 min, followed by blocking with 10% heat inactivated sheep serum for 2 h. The probe-target complex was detected immunologically by a digoxigenin antibody conjugated to alkaline phospatase acting on nitro blue tetrazolium chloride/5-bromo-4-chloro-3-indolyl phosphate (NBT/BCIP, Roche Applied Science, Mannheim, Germany) according to the manufacturer’s protocol. Slides were examined under a light microscope.

### Statistics

Data are presented as means ± SEM for at least three separate experiments (each in triplicate or duplicate). An unpaired Student’s *t*-test was used for statistical analysis. For multiple group comparisons, ANOVA and Bonferroni *t-*test were used. A *P* value of <0.05 compared with control was considered statistically significant.

## Conclusion

Upregulation of GPR40 enhances the mitogenic response to EETs, and a relatively high expression level of GPR40 occurs selectively in a subset of renal tubules including cortical collecting ducts in the mammalian kidney. However, future studies are required to determine whether GPR40 serves as a functional receptor for EETs to mediate EETs’ biologic effects.
